# Terahertz Imaging for Breast Cancer Detection

**DOI:** 10.3390/s21196465

**Published:** 2021-09-28

**Authors:** Lulu Wang

**Affiliations:** 1 Biomedical Device Innovation Center, Shenzhen Technology University, Shenzhen 518118, China; wanglulu@sztu.edu.cn; 2 Institute of Biomedical Technologies, Auckland University of Technology, Auckland 1010, New Zealand

**Keywords:** terahertz radiation, radiation source, terahertz imaging system, terahertz imaging

## Abstract

Terahertz (THz) imaging has the potential to detect breast tumors during breast-conserving surgery accurately. Over the past decade, many research groups have extensively studied THz imaging and spectroscopy techniques for identifying breast tumors. This manuscript presents the recent development of THz imaging techniques for breast cancer detection. The dielectric properties of breast tissues in the THz range, THz imaging and spectroscopy systems, THz radiation sources, and THz breast imaging studies are discussed. In addition, numerous chemometrics methods applied to improve THz image resolution and data collection processing are summarized. Finally, challenges and future research directions of THz breast imaging are presented.

## 1. Introduction

Breast cancer is the leading cause of female cancer death in the United States [1]. Early breast cancer detection with treatment would significantly reduce associated mortality [2]. Approximately 70% of breast cancer patients undergo breast conservation surgery (BCS) [3], and about 15–20% of BCS requires recurrence [4]. Accurately diagnosing tumors is essential for complete resection and minimizing surgeries. Physical examination, X-ray mammography, ultrasound imaging, magnetic resonance imaging (MRI), and positron emission tomography (PET) are the most commonly available clinical modalities for breast tumor detection [5]. Although X-ray mammography is the current standard breast imaging method, it produces harmful ionizing radiation and high false-negative rates and is unsuitable for dense breasts or young women [6]. Ultrasound imaging can produce real-time breast images without cause ionizing radiation but has relatively low image resolution [7]. MRI employs strong magnetic fields and radiofrequency waves to create a picture of internal organs. The image resolution depends highly on magnetic field induction, which defines the main drawback of MRI systems related to expensive magnets [8]. PET detects low ionizing radiation produced by the small number of radioactive materials injected into a patient [9]. Developing a new noninvasive imaging technique to detect breast tumors accurately is urgently needed.

Terahertz (THz) waves (0.1–10 THz) have various features, such as producing low power levels [10], being nonionizing [11], offering good penetration capabilities and resolution [12], and having sensitivity to water molecules [13]. Based on these unique features, THz radiation has potential applications in biology [14], security [15], data communication [16], biomedical imaging [17,18], and food and agriculture [19]. Over the past two decades, THz imaging and spectroscopy have been investigated for biomedical applications [20,21], including diagnosis of liver cirrhosis [22], burn wounds [23], breast cancer [24], colon tumor [25], brain tumor [26], skin cancer [27], and other diseases [28,29,30].

Over the past decade, many research groups worldwide have extensively studied THz imaging and spectroscopy techniques for identifying breast tumors. For rapid imaging, a portable THz pulsed imaging (TPI) system has been applied to analyze basal cell carcinoma (BCC) ex vivo and in vivo [31], and it has been studied for imaging breast tumors [32]. Ashworth et al. employed THz pulsed spectroscopy to study freshly excised human breast cancer tissues [33]. Bowman et al. conducted a comparison study of THz transmission and THz reflection imaging for analyzing the characterization of excised breast carcinomas [34]. The results showed clear differentiation between cancer, collagen, and fat tissues. They also studied the feasibility of using THz imaging to identify three-dimensional dehydrated breast cancer [35] and freshly excised murine tumors [36]. Grootendorst et al. investigated the feasibility of using a handheld TPI system to differentiate benign and malignant breast tissue [37]. Recently, Cassar et al. conducted a pilot study of freshly excised breast tissue using a TPI system over the frequency range from 300 to 600 GHz [38]. THz imaging has the potential to detect tumors in breast-conserving surgery (BCS) accurately.

Numerous chemometrics approaches have been developed for THz applications to produce a good correlation with pathology counterparts. Liu et al. proposed several classifiers, including support vector machine (SVM), k-nearest neighbor, and ensemble learning, to identify breast invasive ductal carcinoma (IDC) using a transmission THz system [39,40,41]. Artificial neural network (ANN) [42], deep convolutional neural network [43], and other approaches [44,45,46,47,48,49] have been proposed and applied in THz imaging and THz-TDS to analyze and enhance THz signals.

This paper summarizes the recent development of THz imaging for breast cancer detection. This article also presents numerous chemometrics methods applied to improve THz image resolution and data collection processing. The research findings demonstrated that THz technologies have the potential to diagnose breast cancer.

## 2. Dielectric Properties of Breast Tissue

The dielectric properties (relative permittivity, conductivity) of biological tissue are highly dependent on frequency [50]. Increasing frequency leads to a decrease in relative permittivity but an increase in conductivity. The single-Debye model demonstrates the complex permittivity of human tissue in a very low frequency range [51]. The dielectric model can characterize the complex permittivity of tissues, which reflects the interaction between molecules and THz radiation [52]. For example, the double-Debye model could demonstrate the complex permittivity of human skin in the THz regime [53]. The Debye model is not suitable for describing tissues that contain less water than skin [54]. The two Debye dispersion relations could accurately predict the dielectric responses of human skin with 70% of water [55]. Dielectric properties of biological tissues with low water content and more complicated structure and composition exhibit broader dispersion that may involve the superposition of several relaxation processes or non-first-order kinematics of molecular structure. The Cole–Cole model could describe the dielectric properties of human tissues [56].

**Figure 1:**
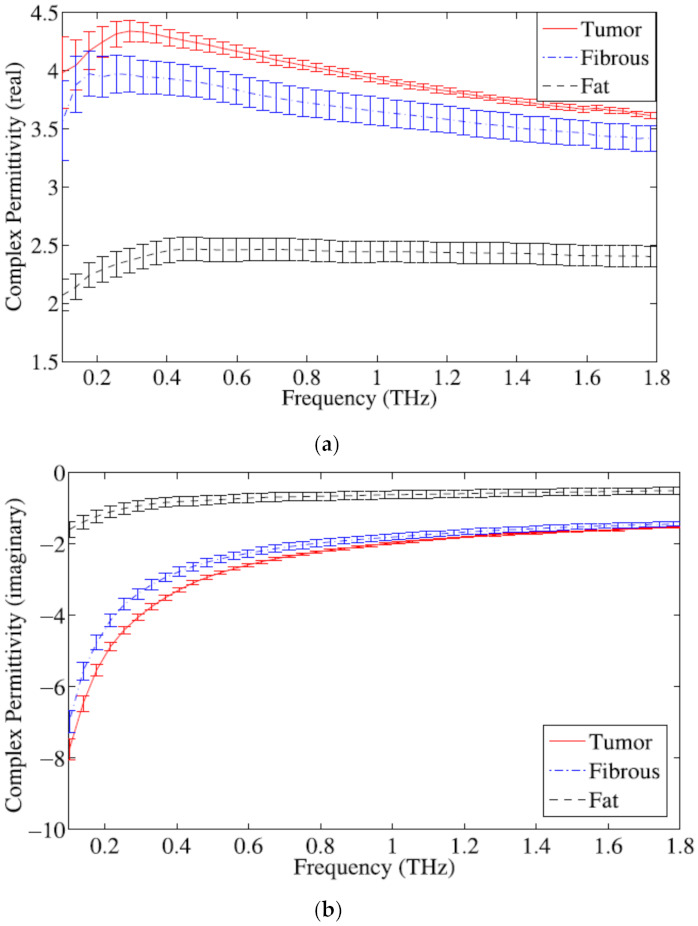
Complex permittivity of breast tissues: (**a**) real part; (**b**) imaginary part [57].

**Figure 2:**
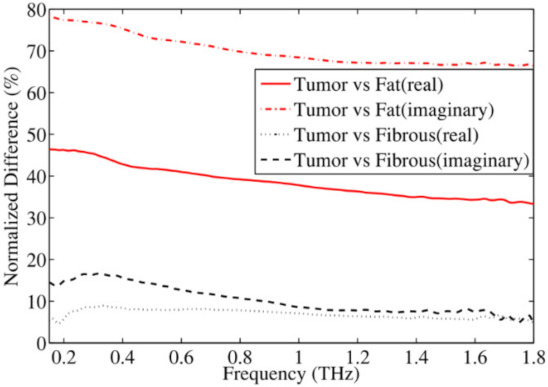
Normalized percentage difference in the average complex permittivity between two breast tissue groups (fibrous and fat) and tumor [57].

Breast tissue contains fat cells and proteins. Fat tissue contains low water that plays a substantial role in regulating dielectric responses of breast tissue. The permittivity of breast tissue increases at lower frequencies and poses a fairly flat response over the higher frequency range. Truong et al. proposed a sum of squared error algorithm to present complex permittivity of breast tissue by combining non-Debye and Debye relaxation processes [57]. Figure 1 shows real and imaginary parts of the complex permittivity of the tumor, fibrous tissue, and fat tissue. Figure 2 shows the normalized percentage difference in the average complex permittivity between fibrous, fat, and breast tumor tissues. Refractive indices and absorption coefficients are measured to calculate the complex permittivity of breast tissues. Pathologists determine the percentage of fat, fibrous, and breast cancer tissues for each sample.

## 3. THz Imaging and Spectroscopy

In 1976, Hartwick et al. investigated far-infrared images using an optically pumped molecular THz laser [58]. Almost 20 years later, in 1995, Hu et al. developed a new chemical imaging system based on optoelectronic THz time-domain spectroscopy [59]. In this study, THz time-domain waveforms were downconverted from THz to kHz frequency range, and the waveform for each pixel was frequency analyzed in real time with a digital signal processor to extract compositional information at that point. The experimental results demonstrated that this system could image biological objects.

Compared to traditional imaging modalities, THz imaging has higher spatial resolution and penetration, which is helpful to identify and analyze nontransparent materials and realize the nondestructive testing of samples. THz imaging systems can be classified as continuous wave (CW) THz imaging and THz pulsed imaging (TPI) based on the THz waveform. CW THz imaging works at a single frequency, while TPI works at a broadband frequency. Detectors in the TPI system can convert THz signals into electrical signals, transform them into an image through the image processing unit, and integrate this information to produce a two-dimensional image.

**Figure 3:**
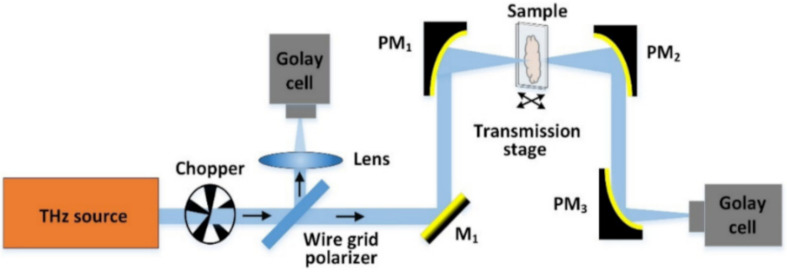
An example of the CWI system [60].

The CW THz imaging systems can be classified as transmission imaging and reflection imaging. The CW THz transmission imaging system is more suitable to map thin tissue samples with weak absorption of THz radiation. As shown in Figure 3, the leaf sample is placed at the focal position of the incident beam and scanned pixel by pixel by moving it along the X–Y plane [60]. It is suitable for verifying the effectiveness of the CW THz transmission imaging system as the leaf can be considered as a 2D thin sample. A few new imaging technologies, such as near-field THz imaging and computer-aided THz tomography, have been proposed for biomedical applications. Table 1 compares the CW THz imaging system with the TPI system [61].

## 4. THz Radiation Sources

**Figure 4:**
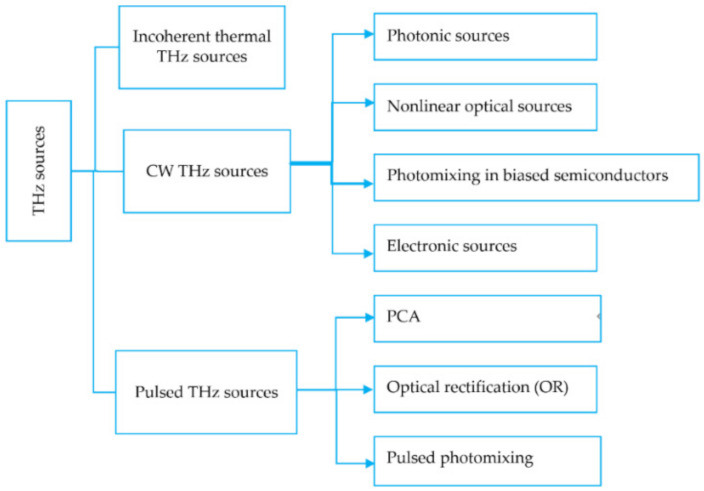
THz sources.

THz radiation sources include incoherent thermal THz sources, CW THz sources, and pulsed THz sources (see Figure 4). CW THz sources can be further divided into four types: photonic sources, nonlinear optical sources, photomixing in biased semiconductors, and electronic sources. Pulsed THz sources can be classified as photoconductive antennas (PCAs), optical rectification (OR), and pulsed photomixing. PCAs use the transient current due to high-speed photoconductors across the radiating antenna. Table 2 compares CW THz radiation source with pulsed THz radiation source [61]. The generation of CW THz radiation depends on the laser. Finding a cost-effective medium that can pump more resourcefully with high gain and power plays a crucial role in developing THz lasers. For example, nonlinear optical material can act as a THz radiation source. High-speed electronic devices can be applied to generate low-power CW THz sources. For instance, complex impedance bridges can be used in the CW THz system to characterize the dielectric property of thin films over the frequency range of 30 GHz to 1 THz. Several mechanisms and techniques, such as photocarriers’ acceleration in PCAs [62], nonlinear optical (NLO) crystals [63], plasma oscillations [64], and nonlinear electronic transmission lines [65], have been investigated to generate pulsed THz radiation.

## 5. THz Imaging for Breast Cancer Detection

THz cancer imaging aims to distinguish cancerous and peritumoral tissue from normal tissue with clear boundaries. The penetration depth of THz waves in human tissue is limited to the water absorption depth. Existing THz breast cancer imaging techniques focus on imaging excised tissue or using a reflection imaging system to study the surface layer of tissue. THz imaging techniques are developed based on water content differences, which can be considered a reference instead of specific identification. Table 3 demonstrates various THz imaging techniques for breast cancer detection.

In 2003, Berry et al. investigated the optical properties of human tissue at THz frequencies using a TPI system over the frequency range from 0.5 to 2.5 THz [66]. The authors studied various tissues, including skin, adipose tissue, striated muscle, artery, vein, nerve following vascular surgery, and blood samples from volunteers. Results suggested that THz imaging has potential for biomedical applications.

**Figure 5:**
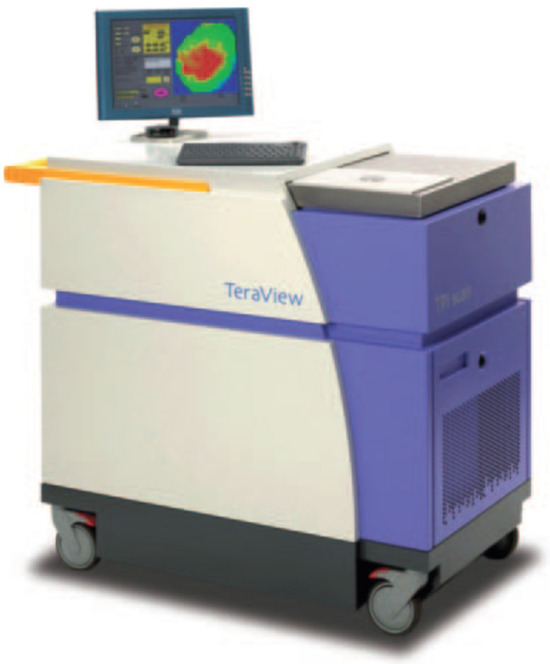
A photograph of the TPI system (TeraView Ltd., Cambridge, UK) [31].

In 2004, Wallace et al. studied the possibility of TPI for discriminating basal cell carcinoma (BCC) and healthy tissue in vivo and ex vivo [31]. In their study, the authors used the TPI scanner (TeraView, see Figure 5) to image 18 BCCs ex vivo and 5 in vivo. The dielectric properties of BCC at THz regions are different from those of healthy tissue. Areas of disease identified in the THz image correlated well with histology. Compared to existing medical imaging approaches, TPI could identify skin cancer and the extent of the neoplasm invasion into the skin with a depth of 250 um. TPI could determine the size of BCC in vivo and delineate cancerous tissues. The research results demonstrated that TPI could help delineate cancerous tissues before surgery. However, further clinical evaluations are required.

**Figure 6:**
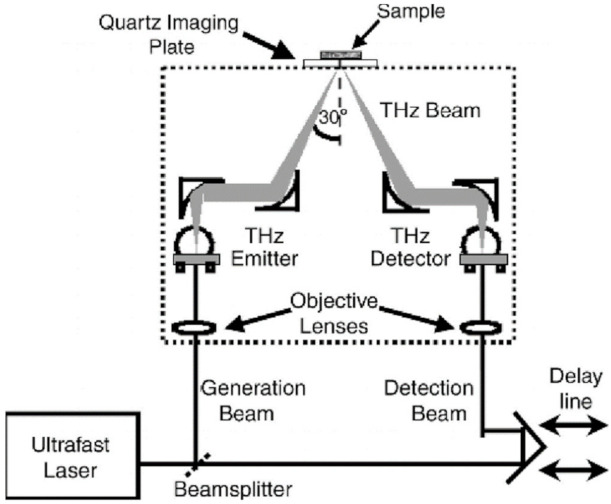
Schematic illustration of a TPI system for imaging breast tissue [32].

In 2006, Fitzgerald et al. studied the feasibility of using TPI to image margins of human breast tumors from 22 nonconsecutive female patients with an average age of 59 years [32]. The authors used the TPI scanner (TeraView Ltd., Cambridge, UK) to map breast specimens. This system uses photoconductive approaches to generate and measure THz pulses in reflection mode. The THz optics (dashed box in Figure 6) focus the beam on the quartz window. The THz optics and the THz beam were rasters scanned in the X–Y plane to record an image set. Two image parameters were investigated: the minimum of the THz impulse function (Emin) and the ratio of the minimum to the maximum of the THz impulse function (Emin/Emax). The correlation coefficient of tumor regions on THz images was comparable with that on photomicrograph. TPI could depict invasive breast carcinoma and ductal carcinoma in situ (DCIS) under control conditions.

In 2009, Ashworth et al. studied characteristics of freshly excised breast specimens from 20 nonconsecutive female patients using a portable THz pulsed transmission spectrometer with an operating frequency of 0.15–2.0 THz [33]. The research results indicated that the THz pulsed spectrometer could identify cancer from fatty and fibrous tissue. In 2011, Chen et al. demonstrated in vivo THz breast cancer imaging in a subcutaneous xenograft mouse study using a compact THz transmission imaging system at room temperature [67]. The THz transmission imaging could distinguish cancer from susceptible surrounding fatty tissue. The detection limit for cancerous tissue is 0.05 mm^3^. Their study is broadly encouraging, and further investigations on early cancer detection using the THz imaging system are required. In the same year, Chen et al. used the CW THz transmission imaging system to investigate 46 different breast cancer specimens and sections without tumors. The absorption coefficients of breast cancer specimens were higher than 9 mm^−1^, and the absorption coefficients of areas without breast tumors were all under 9 mm^−1^ [68]. Results indicated that the CW THz transmission imaging system could distinguish breast tumors from normal tissues but could not identify different types of breast cancer.

**Figure 7:**
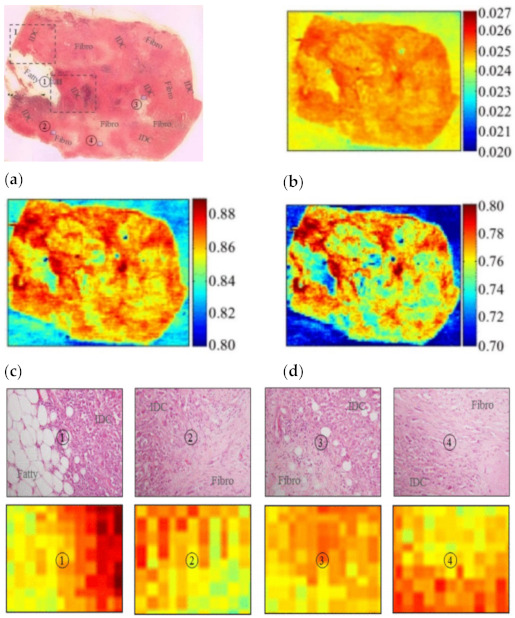
(**a**) Low-power pathology image, ①: regions between fatty and IDC; ②, ③, ④: regions between IDC and fibro. (**b**) THz time-domain image. (**c**) THz frequency-domain photo at 1.5 THz. (**d**) THz frequency-domain shot at 2.0 THz [71].

**Figure 8:**
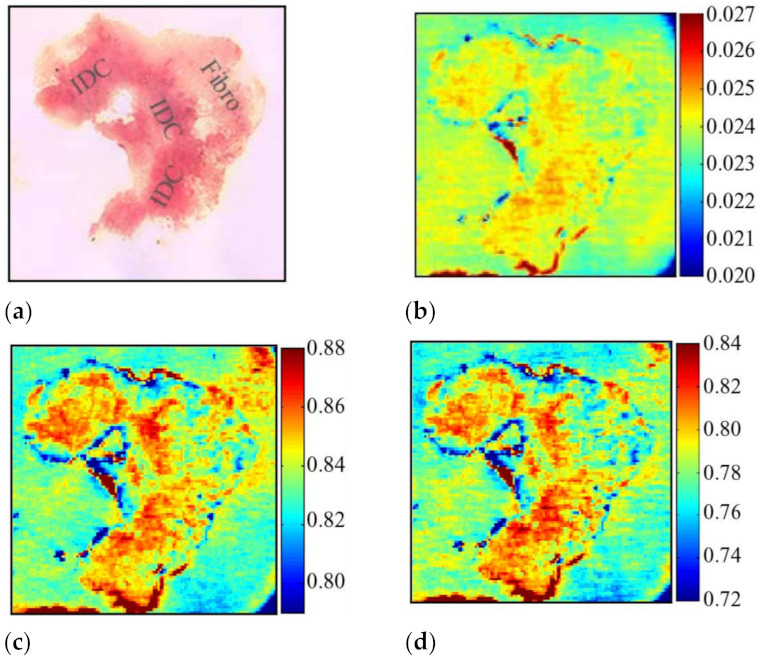
Images of sample 2: (**a**) low-power pathology image used for correlation; (**b**) THz time-domain image; (**c**) frequency-domain image at 1.5 THz; (**d**) frequency-domain image at 1.75 THz [71].

**Figure 9:**
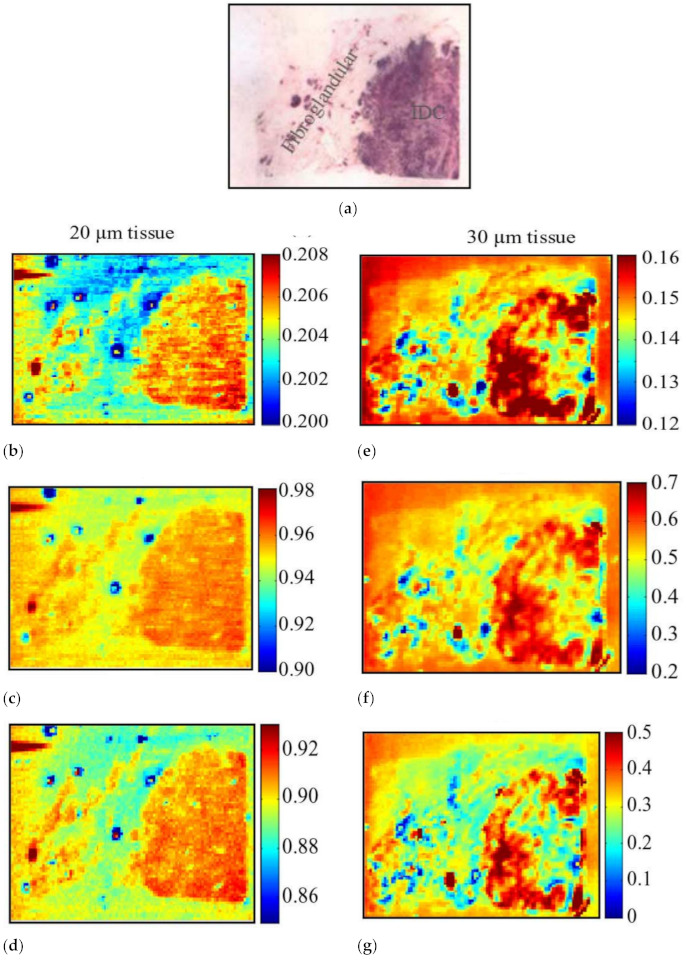
THz image of sample 3. (**a**) Low-power H&E pathology image used for correlation. THz images for 20 µm: (**b**) time-domain image; (**c**) frequency-domain image at 1 THz; (**d**) frequency-domain image at 1.25 THz. THz images for 30 µm: (**e**) time-domain image; (**f**) frequency-domain image at 1 THz; (**g**) frequency-domain image at 1.25 THz [71].

In 2013, Peter et al. applied the reflective THz raster scanning imaging on the breast cancer tissue samples with thicknesses greater than 3–5 mm [70]. They located cancerous areas in excised human breast tissue samples at 1.89 THz and obtained the absolute refractive index values of the breast cancer. In 2015, Bowman et al. investigated the capability of pulsed THz imaging technology for imaging and analysis of heterogeneous breast cancer tissue [71]. The specimens were obtained from breast tumors diagnosed as triple-negative IDC. All flat tissue sections were fixed in formalin, embedded in paraffin, and cut into sections with three thicknesses: 10, 20, and 30 µm. These tissues were classified into three samples. Figure 7 displays images of sample 1, 10 µm thickness tissue obtained from a 40-year-old Caucasian female via mastectomy. Figure 7a shows the macroscopic low-power histopathology image, which denotes the general regions of the tissue types observed in this sample using the stained slide. Figure 8 shows images of sample 2, 10 µm thickness tissue obtained from a 46-year-old Caucasian female via mastectomy. Figure 8a shows a low-power pathology image of the H&E-stained slide. This image further defines regions of IDC and fibrous tissue identified by the pathologist. Figure 8b shows the time-domain image with a color bar scale of the deconvolved electric field amplitude ranging from 0.02 to 0.027. Figure 8c,d display frequency domain images at 1.5 and 1.75 THz, respectively. The results demonstrated that THz reflection imaging could distinguish between the heterogeneous regions in the tumor. Figure 9 shows THz images of sample 3, 20 and 30 µm thickness tissues obtained from a 54-year-old Black woman diagnosed with IDC.

**Figure 10:**
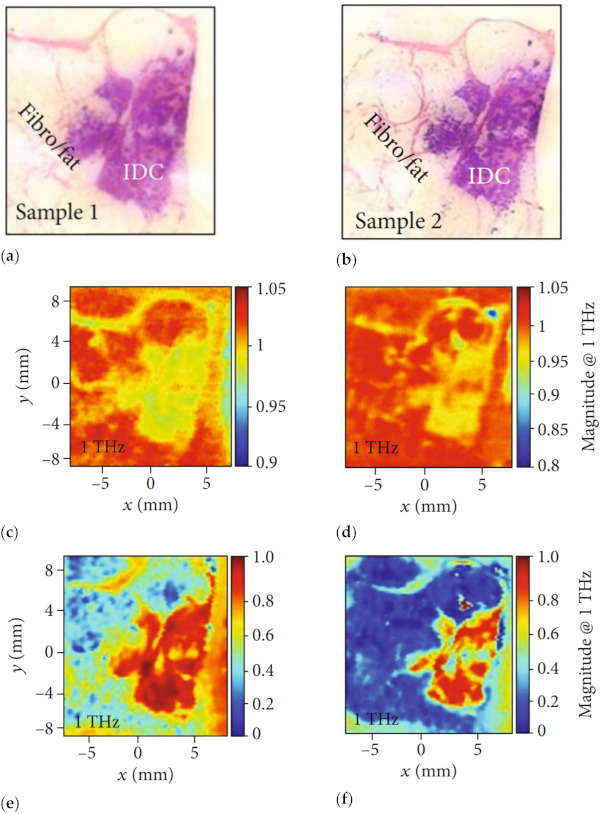
Images of IDC. Pathology of (**a**) sample 1 and (**b**) sample 2. Transmission magnitude images at 1 THz of (**c**) sample 1 and (**d**) sample 2. Reflection magnitude images at 1 THz of (**e**) sample 1 and (**f**) sample 2 [34].

In 2016, Bowman et al. conducted a comparison study of THz transmission and reflection imaging for imaging excised breast carcinomas [34]. Figure 10 shows images of IDC at 1 THz, where cancerous areas exhibited lower transmission and higher reflection than the adjacent healthy areas of fibrous and fatty tissues. The contrast is attributed to higher water content in cancerous areas, which resulted in higher absorption, reflection, or refractive index with THz waves. Compared to transmission imaging, the THz reflection imaging provides higher resolution and more apparent margins between cancerous and fibrous tissue, cancerous and fat tissue, and fibrous and fat tissue regions.

In 2017, Grootendorst et al. investigated the feasibility of using TPI for detecting breast cancer from benign breast tissue in ex vivo [37]. A total of 257 pixels measured from 46 breast tissue samples were imaged using a handheld THz pulsed imaging system for analysis. The images were classified using two data analysis and classification methods. The first method was based on heuristic parameters with the SVM, and the second was based on Gaussian wavelet deconvolution with Bayesian classification. The obtained results indicated that the probe could distinguish invasive breast cancer from benign tissue.

In 2018, Cassar et al. investigated the potential of THz imaging for freshly excised breast tissue [38]. Sixteen freshly excised breast tissue samples were collected and analyzed directly after excision. The principal component analysis (PCA) approach was applied to classify tissue automatically. The research findings demonstrated that the dielectric response could contrast breast tissue recognition over 300–600 GHz. In that same year, Bao et al. proposed two concentration analysis methods, namely the effective medium theory (EMT) model and empirical EMT model, to analyze phantoms mimicking breast tissue [74]. The double-Debye model was applied to describe the dielectric properties of breast phantoms. The EMT with the proposed optimization method helped determine the two-component breast tissue phantoms, including intralipid and water–gelatin emulsions. However, this approach is unsuitable for three-dimensional phantoms because it fails to estimate component concentrations of low-water-content tissues. Research findings confirmed that the proposed method has the potential for quantifying breast tissue pathology. However, more experimental evaluation studies are required in the future.

Recently, some new technologies have been introduced for THz biomedical applications. Serita et al. proposed a nonlinear optical crystal-based THz microfluidic chip for ultratrace and quantitative measurements of liquid solutions [78]. This chip consists of a THz radiation source, a single microchannel, and a few split-ring resonators arrays. The proposed THz microfluidic chip opens a new door for developing lab-on-chip devices. The proposed sensor can detect cancer and diabetes using a small body fluid and reduce pain during diagnosis. Balbekin et al. applied the THz pulse time-domain holography to map breast tissue samples [79]. The experimental measurement involved measuring the diffraction pattern of THz pulse wave distribution at some distance behind the object in the time domain, allowing reconstruction of amplitude and phase at the object plane for the wavefront in the spectral domain. A breast biopsy sample containing cancer tissues was applied in the experiment. The authors performed simulations based on experimental setups to validate the image reconstruction method. Zhang et al. developed a THz microfluidic chip to obtain the THz absorption signatures of a linear single-stranded DNA with 60 nucleotides [80]. This research provided a new way to obtain THz spectra of biomolecules in solution.

## 6. Chemometrics Methods in THz Imaging

Recently, numerous chemometrics methods, such as principal component analysis (PCA), partial least square method (PLS), linear discriminant analysis (LDA), decision tree (DT), genetic algorithm (GA), cluster analysis (CA), random forest (RF), partial least squares discrimination analysis (PLS-DA), ANN, and SVM, have been applied in THz imaging to improve THz data collection speed and accuracy. In addition, machine learning techniques have been used to enhance the visualization of THz images [81,82,83,84,85,86,87].

In 2012, Fitzgerald et al. applied the efficacy of data reduction and SVM techniques in TPI images to classify tumor and normal breast tissue [88]. When using 10 components, the best classification accuracy came from using the principal components on the pulses and prominent features on the parameter, with an accuracy of 92%. The results indicated that data reduction and SVM classifier could provide tissue classification accurately.

In 2015, Qi et al. investigated the feasibility of using THz-TDS with SVM and PLS-DA for fast detection of cervical carcinoma [89]. The authors applied several preprocessing methods, including multiplicative scatter correction (MSC), Savitzky Golay (SG) smoothing and first derivative, principal component orthogonal signal correction (PC-OSC), and emphatic orthogonal signal correction (EOSC) for PLS-DA and SVM models. The SVM and PLS-DA combined with SG first derivative and PC-OSC based on THz-TDS tissue could provide a better method for diagnosing cervical carcinoma.

In 2017, Zhang et al. used THz spectroscopy with chemometrics for discriminating traditional herbal medicines in the frequency range of 0.2–1.2 THz [90]. Four classifiers (PCA, SVM, DT, and RF) were studied to differentiate herbal medicines. Experimental results indicated that PCA with RF could quickly distinguish three types of herbal medicines with an accuracy of 99%. The SVM could improve the accuracy of noninvasive detection of breast cancer using THz imaging technology.

In 2020, Liu et al. studied an automatic recognition strategy for THz pulsed signals of breast IDC based on a wavelet entropy feature extraction and a machine learning classifier [91]. The authors proposed the index of energy to Shannon entropy ratio for distinguishing different tissues. The PCA method and three machine learning classifiers (ensemble, kNN, and SVM) were applied to classify THz signals from breast IDC. Results showed that the ensemble classifier has the best breast IDC identification performance with a precision of 92.85%, which offers an effective automatic recognition strategy for THz biomedical applications.

In 2021, Cassar et al. studied the combination of the refractive index and morphological dilation to improve performances towards breast tumor margin delineation during breast-conserving surgeries [92]. In this study, morphological dilation combined with the refractive index approach was applied for tissue classification. Compared to other configurations, incorporating a wide structuring element and a high refractive index improved the correctness of tissue classification. The research results indicated that combining optical properties of tissues denoted by refractive index with morphological dilation may provide a new way to define supporting procedures during breast-conserving surgeries.

## 7. Conclusions

This paper presented the feasibility of using THz imaging techniques for diagnosing breast disease. The dielectric properties of breast tissue, THz imaging and spectroscopy techniques, THz radiation sources, and THz imaging for breast cancer detection were discussed. This paper also discussed the feasibility of using machine learning classifiers to improve THz image resolution. THz imaging has been proposed as the potential for imaging breast tumors during surgeries. However, to date, THz imaging techniques are still not mature. There are some challenges in THz imaging of breast tumors: (1) a standard medical imaging tool is required to provide comparison results; (2) excess fluid around or under tissue will cause inaccurate detection results; (3) shape changes in tissue occur during the histopathology process between THz and pathology; (4) shape changes can be solved using margin marking ink on tissue.

Further developments of THz techniques are required before they can be practically applied for breast cancer detection. Future research should focus on the following areas: (1) investigating the impact of THz radiation on biological tissues; (2) developing a compact, sensitive, and cost-effective THz imaging system that is more suitable for clinical trials; (3) conducting clinical trial studies of THz imaging for human breast; (4) developing more effective drying techniques to reduce excess fluid around or under the tissue; (5) combining THz imaging with THz spectral fingerprinting of biomarkers to realize qualitative identification and quantitative analysis simultaneously; (6) establishing a THz fingerprint database to improve the recognition efficiency and accuracy of THz imaging techniques.

## Figures and Tables

**Table 1:** Comparison between CW THz imaging system and TPI system [61].

Parameter	CW THz Imaging System	TPI System
Cost	USD 50,000–150,000	USD 300,000–1,000,000
System complexity	Data	Data
Data complexity	Low	High
Weight	About 2 kg	About 300 kg
Information	Transmitted energy	Magnitude information; Phase information; Shape of pulse; Transmission time; Absorption spectrum; Depth
Speed	0.005 s per point 1 mm step size	20–0.05 s per waveform

**Table 2:** Generation of CW and pulsed THz radiations [61].

CW THz Radiation		Pulsed THz Radiation	
Generation approach	Medium	Generation approach	Medium
Photomixing	Power cable switch	Transient photoconductive switching	PCAs
Difference frequency generation using parametric oscillation	Nonlinear crystal	OR	Dielectrics, semiconductors, organic materials
Rotational transitions	Far-infrared gas lasers	Emission from a periodically undulated electron beam	Electron accelerators
Streaming motion and population inversion	Semiconductor laser	Surge current	Semiconductor
Frequency multiplication of microwaves	Schottky barrier diode	Tunneling of electron wave packet	Quantum semiconductor structures
Transitions in superlattice	Quantum cascade lasers	Coherent longitudinal optical phonons	Semiconductors, semimetals, superconductors
Electron interactive with a traveling electromagnetic wave	Backward wave oscillator	Optically short-circuiting the switch	High-temperature superconductor bridge
Relativistic electron interaction with transverse magnetic field	Free-electron lasers	Nonlinear transmission line	Electronic circuits consisting of NLTL

**Table 3:** Current THz breast imaging studies.

Year	Frequency	THz System	Target	Results
Berry et al., 2003 [66]	0.5–2.5 THz	TPI system	Various human tissues	Observed significant differences between broadband refractive indices of several tissues
Wallace et al., 2004 [31]	0.1–3.0 THz	TPI scanner (Teraview Ltd., Cambridge, UK)	BCC and healthy tissue	Could identify the extent of BCC in vivo and delineate tumor margins
Fitzgerald et al., 2006 [32]	0.1–3.0 THz	TPI scanner (Teraview Ltd., Cambridge, UK)	Freshly excised human breast tissues	Could depict invasive breast carcinoma and ductal carcinoma
Ashworth et al., 2009 [33]	0.15–2.0 THz	A portable THz pulsed transmission spectrometer	Freshly excised human breast specimens	THz pulsed spectroscopy and TPI could distinguish healthy adipose breast tissue, healthy fibrous breast tissue, and breast cancer
Chen et al., 2011 [67]	320 GHz	CW THz near-field microscopy transmission imaging	Frozen sliced breast tumors	Breast tumor could be distinguished from normal tissue without H&E staining with a resolution of 240 μm
Chen et al., 2011 [68]	108 GHz	Fiber-scanning transmission THz imaging	Subcutaneous xenograft mouse	Detection limit for tumor size reached 0.05 mm^3^
Joseph et al., 2011 [69]	1.39 and 1.63 THz	CW THz transmission imaging	BCC	Observed good contrast between cancer and normal tissues with a spatial resolution of 390 μm at 1.4 THz and 490 μm at 1.6 THz
Peter et al., 2013 [70]	1.89 THz	CW THz imaging mode	Human breast cancer tissue	Observed absolute refractive index values of samples
Bowman et al., 2015 [71]	0.1–4.0 THz	TPS Spectra 3000 model	Paraffin-made breast phantoms	Could detect heterogeneous sample with a thickness of 10 μm
Bowman et al., 2016 [34]	0.1–4.0 THz	TPS Spectra 3000 system	Excised breast carcinomas	Provided higher resolution and more apparent margins between cancerous and fibro, cancerous and fat, fibro and fat
Bowman et. al., 2017 [35]	0.1–4.0 THz	TPS Spectra 3000 system	IDC and lobular carcinoma embedded in paraffin blocks	Tumor detection is accurate to depths over 1 mm.
Bowman et al., 2018 [72]	0.5–1.0 THz	THz reflection mode	Freshly excised breast tumors	Achieved good agreement between THz and pathology images
Grootendorst et al., 2017 [37]	0.1–1.8 THz	TPI handheld probe system (Teraview Ltd., Cambridge, UK)	Freshly excised breast cancer samples	Could discriminate breast cancer from benign tissue with an encouraging degree of accuracy
Chernomyred et al., 2018 [73]	10.6 THz	CW THz SI microscopy reflectivity imaging system	Human breast specimen	Observed a fragment of the stroma of breast ex vivo
Cassar et al., 2018 [36]	300–600 GHz	TPI and spectroscopy	Freshly excised murine xenograft breast cancer tumors	Cancerous identification accuracy of 80%
Bao et al., 2018 [74]	0.06–4.0 THz	TeraPulse 4000 system (Teraview Ltd., Cambridge, UK)	Freshly excised breast tissue	Spatial resolution reached 1 mm
Vohra et al., 2018 [75]	0.1–4.0 THz	TPI system with a reflection mode (Teraview Ltd., Cambridge, UK)	Freshly excised and formalin/paraffin-fixed breast tumor tissues from a mouse model	Cancerous areas exhibited the highest reflection and agreed with the pathology results
Okada et al., 2019 [76]	~~	A scanning laser THz near-field reflection imaging system	Paraffin-embedded human breast	Spatial resolution reached 20 μm
Bowman et al., 2019 [77]	0.5–1.0 THz	TPS Spectra 3000 pulsed THz imaging and spectroscopy system (Teraview Ltd., Cambridge, UK)	Freshly excised breast cancer tumors	Cancerous areas exhibited higher absorption coefficients and refractive indexes than normal tissues, and the resolution reached 200 μm

## Data Availability

Not applicable.
